# Do *APOE*4 and long COVID-19 increase the risk for neurodegenerative diseases in adverse environments and poverty?

**DOI:** 10.3389/fnins.2023.1229073

**Published:** 2023-08-24

**Authors:** Gabriella C. V. Ciurleo, José Wagner Leonel Tavares-Júnior, Carlos Meton A. G. Vieira, Pedro Braga-Neto, Reinaldo B. Oriá

**Affiliations:** ^1^Laboratory of the Biology of Tissue Healing, Ontogeny and Nutrition, Department of Morphology and Institute of Biomedicine, School of Medicine, Federal University of Ceará, Fortaleza, Ceará, Brazil; ^2^Neurology Division, Department of Clinical Medicine, Faculty of Medicine, Federal University of Ceará, Fortaleza, Ceará, Brazil; ^3^Health Sciences Center, State University of Ceará, Fortaleza, Ceará, Brazil

**Keywords:** long COVID-19, Alzheimer's disease, antagonistic pleiotropy, apolipoprotein E, malnutrition, poverty

## Introduction

In 2020 Global Poverty Monitoring saw the largest one-year increase since its beginning, with an increase of 11% of people living below extreme poverty (from 648 million to 719 million). The economic impact brought by COVID-19, apace with ongoing wars and conflicts, has created a bigger setback toward progress. Low income countries (LIC) and lower middle income countries (LMIC) suffered the highest increases in extreme poverty due to restrictions, as many households, already impoverished before the pandemic, lost their income (Josephson et al., 2021; World Bank, 2022). World hunger increased in 2021, affecting at least 702 million people, and a worrisome issue is inflation in consumer food prices, partly due to various degrees of food supply chain disruption and economic crisis, making it a challenge to afford a healthy diet (FAO et al., 2022).

Worldwide, one billion children under the age of 5 years are affected by parasitic intestinal infection due to living in poverty and developing malnutrition and diarrhea, which is still a public health concern in LIC and LMIC groups (Nantege et al., 2022; Tareke et al., 2022). This is primarily due to inadequate housing, unsafe drinking water, poor sanitation, severe and repeated infections, poor diet, and limited access to healthcare (Pinkerton et al., 2016; Ghosh et al., 2021). Environmental enteropathy and enteric infections owing to recurrent conditions of poor sanitation, culminating with malnutrition, lead to persistent gut barrier disruption and inflammation abrogating the individual‘s capacity to adequately absorb nutrients and oral vaccines with impaired gut immune responses (Guerrant et al., 2013; Gilmartin and Petri, 2015; Tshala-Katumbay et al., 2015).

Epigenetic mechanisms may retain the developmental plastic responses to the environment and orchestrate the evolving phenotype and their stability throughout life (Wang et al., 2014). Certain genetic traits may favor better adaptations for thriving in adverse environments and display a trade-off cost during lifespan with long-lasting implications in growth development, cognition, and educational performance and increased risk for neurodegeneration and metabolic diseases later in life (Guerrant et al., 2013; Tshala-Katumbay et al., 2015).

Evidence suggests a protective role for the apolipoprotein *E*4 (*APOE*4) allele against heavy burdens of diarrhea in weaning-age children living in adverse environments and poverty in developing countries (Wright et al., 2003; Mitter et al., 2012). Apolipoprotein E (apoE) is a carrier protein that delivers cholesterol to be metabolized for lipid homeostasis and is produced in several tissues and cells, such as the liver, skin, macrophages, and brain (Mahley and Rall, 2000). ApoE binds to cholesterol in the brain and incorporates into membrane structures and myelin, playing an important role in brain development (Göritz et al., 2002). The human apolipoprotein E gene (*APOE*) is polymorphic and harbors three common alleles: *APOE*2, *APOE*3, and *APOE*4, coding distinct isoforms with changes in amino acids at position 112 and 158, which influences the protein's function and stability (Giau et al., 2015).

In this opinion paper, we summarized the updated literature on the pros and cons of harboring *APOE*4, in the context of adverse environments. In addition, we highlight the possible effects of long-COVID-19 and APOE4 as potential risk factors for acquiring neurodegenerative diseases and call attention to the importance of early and precise interventions to avert health complications and hospital costs in public health.

## *APOE*4 and cognitive decline under adverse environments

A model proposed by Dahlgren and Whitehead categorizes health determinants into layers, with general socioeconomic, cultural, and environmental conditions being the outer layer, followed by living and working conditions that also consider water and sanitation, housing, and health care services, the next layer is social and community networks, then lifestyle and the last layer being biological factors (Dahlgren Gand, 1991). It is now well-established that all layers are interconnected and interact with one another to influence an individual's wellbeing (Lawrance et al., 2022).

Although *APOE*4 has not been associated with cognitive development in pediatric populations in resource-rich countries (Turic et al., 2001), *APOE*4-positive children living in adverse environments and afflicted by heavy diarrhea burdens early in life displayed better cognitive outcomes than non-carriers in neuropsychological assessments (Oriá et al., 2010). Additionally, a study with Amazonian forager-horticulturalists revealed that adult *APOE*4 carriers in a highly helminthic-endemic environment showed better cognitive performance when compared to non-carriers. In this population, 85.9% of adults had eosinophilia, used as a proxy of helminth infections, and *APOE*4 status was associated with lower eosinophil and leukocyte counts, suggesting more endurance to infections. In the absence of eosinophilia, however, there was a trend toward cognitive decline (Trumble et al., 2017).

Furthermore, undernourished *APOE*4 targeted replacement (TR) mice infected with *Cryptosporidium parvum*, a protozoon commonly associated with diarrhea in children living in endemic areas of malnutrition, showed a more regulated inflammatory response and more efficient pathogen elimination with higher levels of Toll-Like receptor 9 (TLR9) transcripts which are mediators of the innate immune system against Cryptosporidiosis (Azevedo et al., 2014). *C. parvum*-infected and undernourished *APOE*4 TR mice displayed higher levels of L-arginine selective cationic protein transporter (CAT-1) in the ileum, and this may promote a better intestinal barrier function, mucosal blood flow and repair (Li et al., 2007; Azevedo et al., 2014). Altogether, these findings suggest that *APOE*4 has provided a survival advantage, by improving the gut-brain axis in conditions of environmental enteropathy.

Interestingly, significant differences have been found in metabolite expression and fecal microbial profile between young and old *APOE*4 TR mice, where young mice have a higher phylogenetic diversity and older mice have significantly fewer butyrate-producing bacteria and short-chain fatty acids, which may interfere with metabolism and possibly affect cognitive decline (Tran et al., 2019). This may reflect the antagonistic pleiotropy observed in *APOE*4 and explain in part its protective role in young carriers and its risk factor role for neurodegeneration in older carriers. Antagonistic pleiotropy is a condition when a gene or genetic trait is beneficial for early development and detrimental to aging.

Nonetheless, a study evaluating healthy 12 years old adolescents revealed that although gender was a critical factor when assessing the effects of pollution on metabolic and central nervous systems, *APOE*4 carriers showed higher body mass index (BMI) and glucose levels and decreased cognitive performance (Calderón-Garcidueñas et al., 2016). Although this study focused on healthy children, *APOE*4 alone could impact metabolic and cognitive performance. Furthermore, in adults, this finding harbors a unique public health issue for LIC and LMIC groups, which has been raised before (WHO, 2017).

Cognitively unaffected *APOE*4 carriers between the ages of 20 and 38, from a resource-rich environment, already showed altered functional brain activity during episodic memory encoding and retrieval assessments (Matura et al., 2014). Studies of the early impact of apoE4 on brain activity and functional plasticity in rats have shown that there are structural, molecular, and functional modifications in the different hippocampal regions promoting neuronal constriction and interrupting learning processes, and this may prompt later apoE4-related pathologies (Har-Paz et al., 2021).

This is a glimpse at the early effects of *APOE*4, where it no longer harbors strong protection against cognitive development. As individuals age, with environmental exposure and lifestyle behaviors, apoE4 could discretely compound adverse effects that later trigger neurodegeneration. If that same individual is exposed to other diseases, such as COVID-19, with cognitive implications later in life, it may lead to the onset or aggravate neurodegeneration. The antagonistic pleiotropy of *APOE*4 is a matter of debate and it is unclear whether an *APOE*4 carrier living in an adverse environment and poverty, possibly having encountered prolonged malnutrition, will be at a greater risk of developing neurodegenerative diseases.

## *APOE*4, COVID-19, and neurodegenerative diseases

The brain is the second most prominent site of apoE synthesis in humans, produced mainly by astrocytes and microglia (Boyles et al., 1985). This association is significant, as the *APOE*4 allele confers a higher risk of sporadic Alzheimer's disease (AD) (Liu et al., 2014). A single *E*4 allele increases the risk of AD by 2–3 times, and homozygosity for this allele increases the risk by 5–15 times (Farrer et al., 1997). Likewise, individuals with the *E*2 allele have a 43% reduction in the risk of AD, suggesting a protective role for this allele (Ewbank, 2002).

The mechanism by which this risk arises may involve a greater production of β-amyloid protein (Aβ), this deposit being more severe in the presence of the E4 isoform, as demonstrated in studies with transgenic mice for the gene, in addition to the absence of apoE reducing the β-amyloid deposit (Bales et al., 1997; Huang et al., 2017). ApoE4 is associated with cerebral amyloidogenesis by increasing amyloid beta production to a greater extent than other isoforms and increasing tau hyperphosphorylation under stress (Liu et al., 2013; Huang et al., 2017). Furthermore, *APOE* single nucleotide polymorphisms (SNPs) rs429358 and rs7412 are linked with ischemic cerebral infarction contributing to cerebrovascular diseases in the pathophysiology of many cases of dementia (Pendlebury and Rothwell, 2019; Wu et al., 2020).

A study in the United Kingdom revealed that *APOE*4 status was correlated with severe COVID-19 outcomes. *APOE* gene is highly expressed in the alveolar cells of the lungs, together with angiotensin-converting enzyme 2 (ACE-2) (Kuo et al., 2020). The *APOE*2 allele was found to have a protective role against more severe clinical conditions of COVID-19 in 249 volunteers with a mean age of 49 years (Espinosa-Salinas et al., 2022). The apoE4 isoform facilitates the cell viral entry compared to the apoE3, probably due to the more compact structure of the apoE4 and minor spatial interference, thus augmenting the interaction between SARS-CoV-2 spike protein with ACE receptor (Zhang et al., 2022). *In vitro* model studies have highlighted that SARS-CoV-2 infection preferentially activates astrocytes reducing the length of their cytoplasmatic extensions and increasing cytopathogenic effects (Wang et al., 2021).

COVID-19 cognitive impairment has been evaluated by different studies, occurring both in the acute phase and in the post-acute sequelae of COVID-19 syndrome or the so-called long COVID phase (Alemanno et al., 2021; Baig, 2021; Nalbandian et al., 2021; Tavares-Júnior et al., 2022a). 45% of COVID-19 survivors experience at least one long-term unresolved symptom, 4 months after disease onset (O'Mahoney et al., 2023). In addition to COVID-19, other factors may contribute to cognitive complaints in the acute phase of the disease, such as hospitalization, hypoxemia, and delirium (Sartori et al., 2012; Honarmand et al., 2020). To the best of our knowledge, only one publication from our group evaluated cognitive manifestations after COVID-19 and correlated them with polymorphisms of the *APOE* gene (Tavares-Júnior et al., 2022b). In a new article (under review) with more patients, we found a higher frequency of the *E*4 allele in long COVID patients with cognitive decline.

Notably, it has been recognized that Alzheimer's disease patients harboring *APOE*4 are more at risk for high infectivity and worse COVID-19 outcomes (Goyal et al., 2023). In mice, SARS-CoV-2 can cross the blood-brain barrier through adsorptive transcytosis and this is strongly dependent on glycoproteins' sugars of the virus and targeted cell. In a mouse model of Alzheimer's disease, infection with a non-replicating SARS, as well as S1 (ectodomain of the spike protein), increased neuroinflammation. Accumulated beta-amyloid peptide facilitated the S protein binding to ACE2 and viral internalization (Erickson et al., 2023). Although autopsy studies suggest that very small loads of SARS-CoV-2 RNA are found in the postmortem brain, the primary cause of cognitive impairment (or neurological complications from COVID-19) may arise from hypoxia and systemic inflammation (Thakur et al., 2021), which themselves may lead to neurodegeneration.

## Final considerations

In 2019 non-communicable diseases were responsible for 74% of deaths globally. Diseases such as stroke, ischemic heart disease, diabetes mellitus, and liver cirrhosis are taking over the leading causes of death in LIC and LMIC, and the trend indicates that non-communicable diseases will rise further (WHO, 2020), affecting the risk and early onset of neurodegenerative diseases.

Recent breakthrough advances in machine learning and artificial intelligence may be instrumental in screening big databases in LIC and LMIC populations and building predicted disease risk analytics and better interventional roadmaps regarding the influence of *APOE*4 on neurodegenerative diseases in long COVID-19 patients. Asymptomatic testing programs are important in predicting the rise and progression of new diseases that may eventually synergize with other diseases, leading to increased morbidity and/or mortality. There is a need for more precision medicine centers accessible to a population living in poverty and exposed to adverse environments, as they rely on the public health system alone for care.

There is still a gap of knowledge in addressing whether *APOE*4 compounded with COVID-19 (even asymptomatic) would impact brain development in children and adults living in adverse environments and poverty. Caution should be taken as neurodegenerative diseases are complex and multifactorial borne conditions, however, under certain aggravating circumstances, *APOE*4 and COVID-19 may be strong drivers toward neurodegeneration.

In summary, we call for awareness of the potential public health issue of long COVID-19 as a cumulative risk factor for neurodegenerative diseases in *APOE*4 carriers chronically exposed to adverse environments and limited access to healthcare. Embedding genomics into routine public health practice in LIC and LMIC groups will provide accurate information regarding the stratification of molecular markers and assist in early intervention.

## Author contributions

All authors listed have made a substantial, direct, and intellectual contribution to the work and approved it for publication.
